# Protective effects of hydro-alcoholic extract of *Zataria multiflora* on lipopolysaccharide-induced inflammation and oxidative stress in rat liver 

**DOI:** 10.22038/AJP.2023.21914

**Published:** 2023

**Authors:** Zohreh Arab, Hossein Salmani, Narges Marefati, Farimah Beheshti, Akbar Anaeigoudari, Farzaneh Shakeri, Narges Tajmazinani, Mahmoud Hosseini

**Affiliations:** 1 *Applied Biomedical Research Center, Mashhad University of Medical Sciences, Mashhad, Iran*; 2 *Student Research Committee, Jiroft University of Medical Sciences, Jiroft, Iran*; 3 *Department of Physiology, School of Medicine, Baqiyatallah University of Medical Sciences, Tehran, Iran*; 4 *Neuroscience Research Center, Torbat Heydariyeh University of Medical Sciences, Torbat Heydariyeh, Iran*; 5 *Department of Physiology, School of Paramedical Sciences, Torbat Heydariyeh University of Medical Sciences, Torbat Heydariyeh, Iran *; 6 *Department of Physiology,* *School of Medicine, Jiroft University of Medical Sciences, Jiroft, Iran*; 7 *Natural Products and Medicinal Plants Research Center, North Khorasan University of Medical Sciences, Bojnurd, Iran*; 8 *Department of Physiology and Pharmacology, School of Medicine, North Khorasan University of Medical Sciences, Bojnurd, Iran*; 9 *Pharmacological Research Center of Medicinal Plants, Mashhad University of Medical Sciences, Mashhad, Iran*; 10 *Psychiatry and Behavioral Sciences Research Center, Mashhad University of Medical Sciences, Mashhad, Iran*

**Keywords:** Zataria multiflora, Oxidative stress, Inflammation, Lipopolysaccharide, Liver enzyme

## Abstract

**Objective:**

Liver is an important player in regulation of body homeostasis. Study investigated the effects of hydro-alcohol extract of *Zataria multiflora* (ZM) on oxidative damage, level of IL-6 and enzymes of liver in lipopolysaccharide (LPS)-treated rats.

**Materials and Methods:**

The rats were distributed into 5 groups: 1) Control; 2) LPS; and 3-5) ZM-Extract (Ext) 50, ZM-Ext 100, and ZM-Ext 200. ZM-Ext groups received 50, 100 and 200 mg/kg of extract 30 min before LPS. Drugs were injected intraperitoneally. The entire period of this project was 17 days. In first three days, only extract was injected and then, ZM was injected along with LPS.

**Results:**

LPS increased the level of ALT (Alanine aminotransferase), AST (Aspartate aminotransferase ), ALK-P (Alkaline Phosphatase), IL-6, malondialdehyde (MDA), and nitric oxide (NO) metabolites and lowered thiol, superoxide dismutase (SOD) and catalase (CAT) concentration. ZM extract not only reduced ALT, AST, ALK-P, IL-6, MDA, and NO metabolites concentrations but also increased thiol content, and SOD and CAT levels.

**Conclusion:**

Extract of ZM prevented LPS-induced hepatotoxicity. This protective effect was associated with reduction in inflammation and oxidative stress.

## Introduction

The liver as a substantial player in regulation of body hemostasis can neutralize toxins and metabolize drugs (Utaipan et al., 2018 ▶). The hepatocytes injury can strongly affect liver function (Asgharzadeh et al., 2017 ▶). One of the most important factors damaging the hepatocytes is ample generation of inflammatory cytokines (Jaeschke et al., 2000 ▶; Mortazavi et al., 2021 ▶). Over-release of reactive oxygen species (ROS) and reactive nitrogen species (RNS) from macrophages can result in rigorous dysfunction hepatocytes (Wei et al., 2014 ▶). Potent endotoxins such as lipopolysaccharide (LPS) can up-regulate the generation and release of inflammatory mediators and cytotoxic agents (Beheshti et al., 2018 ▶). It has been also confirmed that cytotoxic factors production blockers can abolish LPS-induced liver dysfunction (Zhong et al., 2016 ▶).

*Zataria multiflora *(ZM) a medicinal herb grown in many countries including Iran and Pakistan, has been used as a pacificator of pain, oxidative balancer and reducer of inflammatory mediators from ancient times (Boskabady et al., 2015 ▶; Nakhai et al., 2007 ▶). It has been understood that ZM essential oil ameliorated infection-induced wound by downregulation of inflammatory cytokines including tumor necrosis factor alpha (TNF-α) and interleukin-1β (IL-1β) (Farahpour et al., 2021 ▶). In inflammation and oxidative stress resulted from inhaled paraquat, ZM also reduced malondialdehyde (MDA), TNF-α, IL-17 and increased total thiol, catalase (CAT), and IL-10 in rats (Amin et al., 2021 ▶). Anti-diabetic effects associated with improvement of oxidative stress of ZM extract in rats were also reported (Mahmoodi et al., 2019 ▶). Recently, it has been documented that ZM extract could improve learning, memory, anxiety and depression in LPS-caused inflammatory model in rats (Arab et al., 2020 ▶; Arab et al., 2022 ▶). All these therapeutic effects are attributed to ZM extract compounds such as thymol, carvacrol, apigenin, luteolin, and 6-hydroxyluteolin glycosides (Bialuk et al., 2018 ▶). In an experimental study, carvacrol improved asthma in rats exposed by ovalbumin via reducing the expression of IL-4, IL-5, IL-13, TNF-α and MDA and increasing glutathione (GSH) and SOD (Ezz-Eldin et al., 2020 ▶). Carvacrol also attenuated inflammation in LPS-exposed rats (Salmani et al., 2022 ▶). Arigesavan et al. also reported that carvacrol exerted positive effects on colon cancer resulted from 1,2 dimethylhydrazine (DMH) and dextran sodium sulfate in rats by enhancing the level of antioxidant biomarkers such as GSH, SOD and CAT and decreasing the concentration of oxidant agents including myeloperoxidase (MPO) and nitric oxide (NO) (Arigesavan et al., 2015 ▶). 

Considering these pieces of evidence, the current study was programed to examine the effects of hydro-alcoholic extract of ZM on LPS-induced inflammation and oxidative stress in rat liver. 

## Materials and Methods

**Animals and grouping**

Adult male Wistar rats (8-10 weeks old and 250±10 g) provided from animals’ house and guarded under standard conditions were grouped into five groups (n=8 in each group): (1) Control group: animals received saline instead of LPS and saline+ 0.2% tween instead of ZM extract (2) LPS group: animals were injected with 1 mg/kg LPS and saline+ 0.2% tween instead of the extract ; and (3-5) ZM-Ext groups: the animals were treated respectively with 50, 100 and 200 mg/kg of ZM extract dissolved in saline+ 0.2% tween, 30 min before LPS (Arab et al., 2020). Injections were achieved intraperitoneally (i.p.) (Sheibani et al., 2019 ▶). 

Animal handling was done based on guidelines offered by the Ethical Committee of Animal Research of Mashhad University of Medical Sciences, Mashhad, Iran (IR.MUMS.fm.REC.1397.139). Total period of this project was 17 days. In first three days, only extract was injected. After that in the following days, we injected ZM and 30 min later, LPS was administered. On the 17th day, 2 hr after injection of LPS, the animals were anesthetized (urethane 1.6 gr/kg i.p.) and sacrificed and the blood and liver tissues were collected. Then, liver enzymes and oxidative stress factors (MDA, SOD, Thiol, and CAT), NO metabolite (NO_2_) and inflammatory cytokine IL-6 were measured.

**Preparation of the extract**

ZM was provided from local market in Mashhad city, Iran. Identification number of the plant was determined by an expert botanist in herbarium of Ferdowsi University of Mashhad, Mashhad, Iran (35314(. In order to prepare the extract, ZM was comminuted and then 100 g of it was dissolved in 70% ethanol. After 72 hr, the mixture was passed through a special filter. In the last step, water was removed and the extract was used (Hosseinzadeh et al., 2000b ▶; Ischiropoulos et al., 1992 ▶).

**Biochemical assessments**

The liver tissues of rats were homogenized using phosphate buffer. Then, it was centrifuged and the supernatant was used for biochemical assessments. The levels of oxidative stress indexes including thiol, malondialdehyde (MDA), superoxide dismutase (SOD) and catalase (CAT) and NO metabolites were measured as described in previous studies (Karimi et al., 2018 ▶; Khazdair et al., 2018 ▶). IL- 6 concentration in liver tissue was measured using an ELISA kit (ebioscience Co, San Diego, CA, USA) based on the company’s instruction. The amount of liver enzymes such as alanine aminotransferase (ALT), aspartate aminotransferase (AST), alkaline phosphatase (ALK-P) and total protein and albumin was also measured in blood serum based on procedures declared in our previous works (Beheshti et al., 2019 ▶). 

**Statistical analysis**

Findings are reported as mean±SEM. Differences between mean values of normally distributed data were compared by one-way ANOVA followed by Turkey’s post hoc comparison test. A p<0.05 was considered statistically significant.

## Results

**Effect of** **hydro-alcoholic extract of** ***Zataria multiflora*** **on** **IL-6 of NO metabolites in the liver tissue**

**Figure 1:**
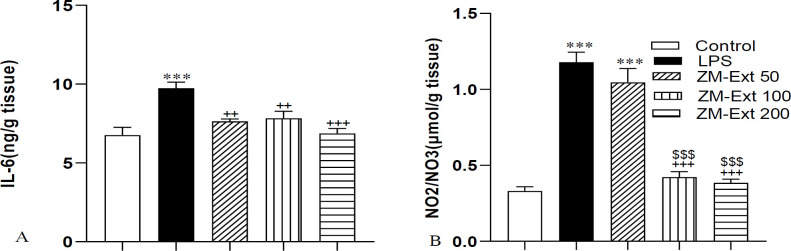
The results of IL-6 (A) and NO metabolites (B) in the liver tissue. The results are illustrated as Mean±SEM. One-way ANOVA was used to analyze data and post hoc Turkey test to compare the groups. LPS increased the level of IL-6 and NO metabolites versus the control group. Extract reversed the effect of LPS. ***p<0.001 compared to the control group, ^++^p<0.01 and ^+++^p<0.001compared to the LPS group, ^$$$^p<0.001compared to the ZM-Ext 50 group

Results indicated that the level of IL-6 in liver tissue of the LPS group was significantly higher than the control group (p<0.001). The concentration of IL-6 in all the ZM-Ext 50, ZM-Ext 100, and ZM-Ext 200 groups was less than the LPS group (p<0.01, p<0.01, and p<0.001, respectively) (Figure 1A). Evaluation of NO metabolites also showed a large amount of this biochemical index in rats liver tissue of LPS the group compared to the control group (p<0.001). The concentration of NO metabolites in the ZM-Ext100 and ZM-Ext200 groups was lower than the LPS group (both p<0.001). In addition, IL-6 concentration in the liver tissue of both the ZM-Ext100 and ZM-Ext200 groups was lower than the ZM-Ext50 group (both p<0.001) (Figure 1B). 

**Effects of hydro-alcoholic extract of** ***Zataria multiflora*** **on** **oxidative stress parameters of liver tissue**

**Figure 2:**
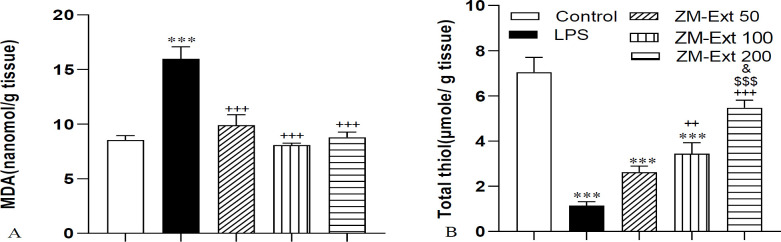
The results of MDA (A) and total thiol (B) in the liver tissue. The results are illustrated as Mean±SEM. One-way ANOVA was used to analyze data and post hoc Turkey test to compare groups. LPS increased the level of MDA and decreased total thiol in comparison with the control group. Extract reversed the effect of LPS. ***p<0.001compared to the control group, ^++^p<0.01 and ^+++^p<0.001compared to the LPS group, ^$$$^p<0.001compared to the ZM-Ext 50 group, ^&^p<0.05compared to the ZM-Ext 100 group

**Figure 3:**
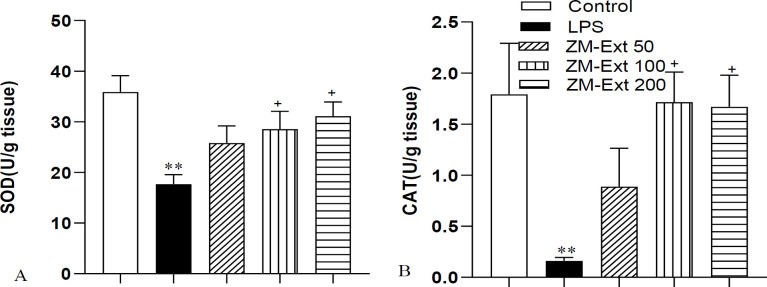
The results of SOD (A) and CAT (B) in the liver tissue. The results are illustrated as Mean±SEM. One-way ANOVA was used to analyze data and post hoc Turkey test to compare groups. LPS reduced the concentration of SOD and CAT in comparison with the control group. Extract increased concentration of SOD and CAT compared to the LPS group. **p<0.01 compared to the control group, ^+^p<0.05 compared to the LPS group

To evaluate lipid peroxidation in the liver tissue of rats, we appraised the MDA concentration. We perceived a high amount of MDA in liver tissue of the LPS-treated rats when it was compared with the control group (p<0.001). The level of MDA in the ZM-Ext 50, ZM-Ext 100, and ZM-Ext 200 groups had a significant decrease in comparison to the LPS group (all p<0.001) (Figure 2A). The findings of the study also exhibited that the level of total thiols in the LPS group was lower than the control group (p<0.001). The total thiol concentration was increased in the ZM-Ext 100 and ZM-Ext 200 groups compared to the LPS group (p<0.01 and p<0.001, respectively). In addition, the total thiol level in the liver tissues of the ZM-Ext200 group was meaningfully higher than that of the ZM-Ext50 and ZM-Ext100 groups (p<0.001 and p<0.05, respectively) (Figure 2B). We also understood that LPS administration decreased the SOD and CAT activity of rats^,^ liver tissue compared to the animals of the control group (both p<0.01). Our findings also revealed a remarkable enhancement in SOD and CAT activity of the ZM-Ext100 and ZM-Ext200 groups compared to the LPS group (both p<0.05) (Figures 3A and 3B). 

**Liver enzymes**

In order to evaluate liver function, the blood level of AST, ALT and ALK-P as well as the concentration of total protein and albumin were measured. Systemic administration of LPS increased the level of AST, ALT and ALK-P compared to the control group (all p<0.001). Administration of 100 and 200 mg/kg of the extract lowered the level of AST in the ZM-Ext100 and ZM-Ext200 groups compared to the LPS group (p<0.01 and p<0.001 respectively). Evaluation of ALT exhibited that the concentration of this hepatic enzyme in all three groups treated by the extract was decreased compared with the LPS group (all p<0.001). The level of ALT in the ZM-Ext200 group also was lower than the Zm-Ext50 and ZM-Ext 100 group (p<0.01 and p<0.05 respectively).

**Figure 4:**
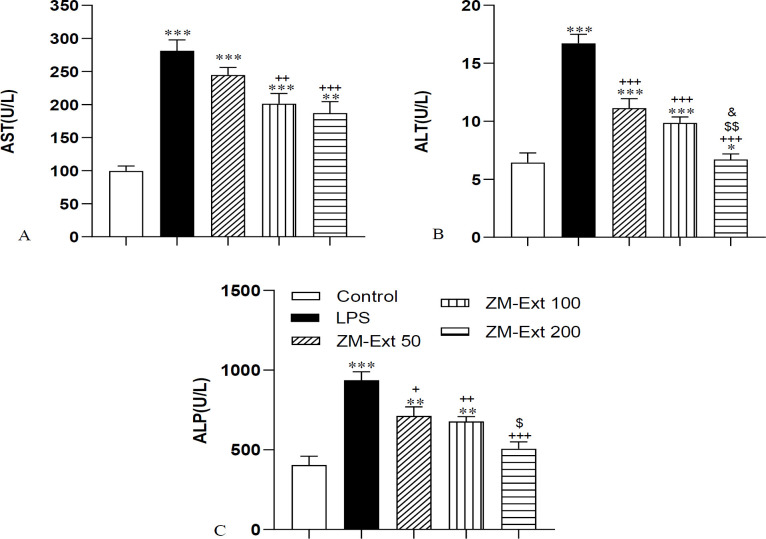
The results of AST (A), ALT (B) and ALK-P (C) in the serum. The results are illustrated as Mean±SEM. One-way ANOVA was used to analyze data and post hoc Turkey test to compare the groups. LPS enhanced the blood concentration of AST, ALT and ALK-P in comparison with the control group. Extract diminished serum concentration of AST and ALT and ALK-P compared to the LPS group. **p<0.01 and ***p<0.001 compared to the control group, ^+^p<0.05, ^++^p<0.01 and ^+++^p<0.001 compared to the LPS group, ^$^p<0.05 and ^$$^p<0.01 compared to the ZM-Ext 50 group, ^&^p<0.05 compared to the ZM-Ext 100 group

**Figure 5:**
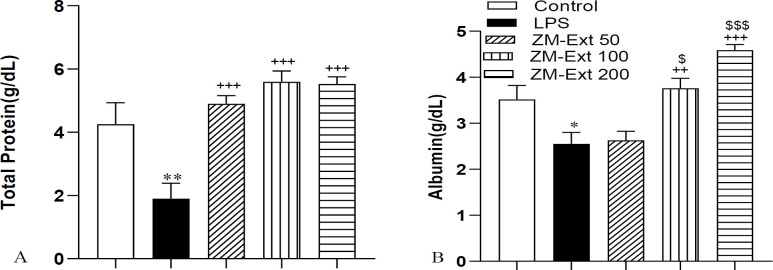
The results of total protein (A) and albumin (B) in the serum. The results are illustrated as Mean±SEM. One-way ANOVA was used to analyze data and post hoc Turkey test to compare the groups. LPS reduced the blood concentration of total protein and albumin in comparison with the control group. Extract increased serum concentration of total protein and albumin compared to the LPS group. *p<0.05 and **p<0.01compared to the control group, ^++^p<0.01 and ^+++^p<0.001 compared to the LPS group. ^$$$^p<0.001compared to the ZM-Ext 50 group, ^&^p<0.05 compared to the ZM-Ext 100 group

The results of ALK-P indicated a decreased concentration of this enzyme in the ZM-Ext50, ZM-Ext100, and ZM-Ext200 groups compared to the LPS group (p<0.05, p<0.01 and p<0.001 respectively). The level of ALK-P in the ZM-Ext200 group was also less than the ZM-Ext50 (p<0.05). Assessment of the results also confirmed a decremented level of total protein and albumin in the LPS group in comparison with the control group (p<0.01 and p<0.05 respectively) (Figures 4A, 4B and 4C). The total protein level in all three groups of extract was higher than the LPS group (all p<0.001). The blood concentration of albumin in the ZM-Ext100 and ZM-Ext200 increased versus the LPS group (p<0.01 and p<0.001 respectively). The level of this plasma protein in the ZM-Ext100 and Zm-Ext200 groups was higher than the Zm-Ext50 group (p<0.05 and p<0.001 respectively) (Figures 5A and 5B).

## Discussion

The liver is considered to have a vital role in omission of toxins and regulation of body homeostasis (Nam et al., 2019 ▶). Based on pathophysiological studies, liver dysfunction is associated with increased serum level of ALK-P, ALT and AST (Hafez et al., 2015 ▶) and decreased concentration of albumin and total protein (Yakubu et al., 2003 ▶). LPS prepared from cell wall of bacterial has also been found to disturb the function of body organs. In an animal study, Beheshti et al. detected that LPS enhanced the blood concentration of ALT, AST and ALK-P and decreased the level of total protein and albumin in rats (Beheshti et al., 2019 ▶). In the present study also the level of ALT, AST and ALK-P was high while the concentration of total protein and albumin was low in blood specimens of the LPS group rats versus the control group. 

Pathophysiological findings emphasize that there is a potent relationship between inflammation and liver diseases including liver fibrosis (Nam et al., 2019 ▶). Researchers reported that injured Kupffer cells can secret inflammatory mediators such as cyclooxygenase-2 (COX2), transforming growth factor beta (TGF-β) and TNF-α (Dinarello, 2000 ▶). It has also been revealed that bacterial and non-bacterial infections can cause hepatocytes dysfunction through increasing the generation of inflammatory cytokines (Pawlotsky, 2004 ▶). Growing evidence shows that systemic administration of LPS stimulates inflammatory responses and triggers the release of IL-1β, IL-6 and TNF-α (Kim et al., 2018 ▶). In this study also hepatic inflammation resulted from peripheral injection of LPS was associated with a significant augmentation in the level of IL-6 in liver tissue of the LPS group animals vs. the control group rats. This finding supports liver inflammation resulting from intraperitoneal administration of LPS in our study. 

Oxidative stress has also been suggested to be as a key factor disturbing normal function of body organs (Anaeigoudari et al., 2016 ▶). Over-accumulation of free radicals and attenuation of antioxidant systems has been recognized to destroy the function of the cardiovascular system (Bhardwaj et al., 2013 ▶), kidney (Sureshbabu et al., 2015 ▶) and brain (Baghcheghi et al., 2018 ▶). Liver tissue has also been shown to be sensitive to oxidative stress (Edenta et al., 2017 ▶). It has been demonstrated that high concentrations of free radicals disturb hepatocytes membrane integrity and upset liver function (Du et al., 2017 ▶). LPS has also been revealed to induce oxidative stress via enhancing ROS production and lipid peroxidation (Azizi-Malekabadi et al., 2015 ▶). In the current study, LPS enhanced oxidant biomarkers including MDA and NO metabolites and reduced antioxidant agents such as total thiol, SOD and CAT in rats’ liver tissue when it was injected peripherally. 

In this work, we examined effects of intraperitoneal injection of hydro-alcoholic extract of ZM on LPS-disturbed liver function in rats. Data indicated that administration of ZM extract before LPS attenuated the harmful effects of this bacterial endotoxin on liver function. There was reduced level of ALT, AST and ALK-P and increased concentration of total protein and albumin in ZM-Ext groups versus the LPS group. 

Scientific findings display that medicinal herbs and their antioxidant and anti-inflammation compounds can exert useful effects on various body systems (Edenta et al., 2017 ▶). ZM is a member of the family Labiatae which has antioxidant, and anti-inflammatory properties (Hosseinzadeh et al., 2000a ▶). The study of Ahmadipour et al. illustrated that ZM extract defended the liver against cisplatin via modulating the activities of ALT, AST, SOD, CAT and glutathione peroxidase in rats (Ahmadipour et al., 2015 ▶). In another study, it has been revealed that hydroalcoholic extract of ZM normalized blood sugar, inflammatory responses, oxidative stress status and liver enzymes in diabetic rats (Mahmoodi et al., 2019 ▶). In a clinical trial, ZM extract also alleviated the clinical symptoms in asthmatic patients by ameliorating oxidative stress and reducing inflammatory cytokines (Alavinezhad et al., 2020 ▶). The positive therapeutic effect of ZM extract on chronic obstructive pulmonary disease (COPD) patients has also been affirmed; in a randomized, doubled-blind clinical trial, ZM extract (3 and 6 mg/kg/day for two months) diminished MDA, nitrite and C-reactive protein levels and augmented total thiol content and SOD and CAT activities and finally improved COPD symptoms (Ghorani et al., 2020 ▶). In the present research also hydroalcoholic extract of ZM normalized the enhanced levels IL-6, MDA and NO metabolites and reduced concentrations of total thiol, SOD and CAT in the ZM-Ext groups compared to the LPS group. In this study besides biochemical measurements, it was better to assess the pathological findings. However, it was not possible for us to do and it is announced as a limitation in our work. In addition, it is recommended to evaluate the oral administration effect of ZM extract in future and compare with the results of this study. 

In summary, we can declare that hydroalcoholic extract of ZM could prevent LPS hepatotoxicity in rats. This protective effect was likely carried out through antioxidant and anti-inflammatory properties of ZM. 

## Conflicts of interest

The authors have declared that there is no conflict of interest.

## References

[B1] Ahmadipour A, Sharififar F, Nakhaipour F, Samanian M, Karami-Mohajeri S (2015b). Hepatoprotective effect of Zataria Multiflora Boisson cisplatin-induced oxidative stress in male rat. J Med Life.

[B2] Alavinezhad A, Ghorani V, Rajabi O, Boskabady MH (2020). Zataria multiflora affects clinical symptoms, oxidative stress and cytokines in asthmatic patient: A randomized, double blind, placebo-controlled, phase II clinical trial. Cytokine.

[B3] Amin F, Memarzia A, Roohbakhsh A, Shakeri F, Boskabady MH (2021). Zataria multiflora and Pioglitazone affect systemic inflammation and oxidative stress induced by inhaled paraquat in rats. Mediators Inflamm.

[B4] Anaeigoudari A, Hosseini M, Karami R, Vafaee F, Mohammadpour T, Ghorbani A, Sadeghnia, HR (2016). The effects of different fractions of Coriandrum sativum on pentylenetetrazole-induced seizures and brain tissues oxidative damage in rats. Avicenna J Phytomed.

[B5] Arab Z, Hosseini M, Mashayekhi F, Anaeigoudari A (2020). Zataria multiflora extract reverses lipopolysaccharide-induced anxiety and depression behaviors in rats. Avicenna J Phytomed.

[B6] Arab Z, Hosseini M, Marefati N, Beheshti F, Anaeigoudari A, Sadeghnia HR, Boskabady MH (2022). Neuroprotective and memory enhancing effects of Zataria multiflora in lipopolysaccharide-treated rats. Vet Res Forum.

[B7] Arigesavan K, Sudhandiran G (2015). Carvacrol exhibits anti-oxidant and anti-inflammatory effects against 1, 2-dimethyl hydrazine plus dextran sodium sulfate induced inflammation associated carcinogenicity in the colon of Fischer 344 rats. Biochem Biophys Res Commun.

[B8] Asgharzadeh F, Bargi R, Beheshti F, Hosseini M, Farzadnia M, Khazaei M (2017). Thymoquinone restores liver fibrosis and improves oxidative stress status in a lipopolysaccharide-induced inflammation model in rats. Avicenna J Phytomed.

[B9] Azizi-Malekabadi H, Hosseini M, Pourganji M, Zabihi H, Saeedjalali M, Anaeigoudari A (2015). Deletion of ovarian hormones induces a sickness behavior in rats comparable to the effect of lipopolysaccharide. Neurol Res In.

[B10] Baghcheghi Y, Beheshti, F, Shafei MN, Salmani H, Sadeghnia HR, Soukhtanloo M, Anaeigoudari A, Hosseini M (2018). The effects of vitamin E on brain derived neurotrophic factor, tissues oxidative damage and learning and memory of juvenile hypothyroid rats. Metab Brain Dis.

[B11] Beheshti F, Hosseini M, Taheri Sarvtin M, Kamali A, Anaeigoudari A (2019). Protective effect of aminoguanidine against lipopolysaccharide-induced hepatotoxicity and liver dysfunction in rat. Drug Chem Toxicol.

[B12] Beheshti F, Norouzi F, Abareshi A, Khazaei M, Alikhani V, Moussavi S, Biglari G, Soukhtanloo M, Hosseini M (2018). Nigella sativa prevented liver and renal tissue damage in lipopolysaccharide-treated rats. Saudi J Kidney Dis Transpl.

[B13] Bhardwaj P, Khanna D (2013). Green tea catechins: defensive role in cardiovascular disorders. Chin J Nat Med.

[B14] Bialuk I, Taranta A, Winnicka MM (2018). IL-6 deficiency alters spatial memory in 4- and 24-month-old mice. Neurobiol Learn Mem.

[B15] Boskabady MH, Gholami Mahtaj L (2015). Lung inflammation changes and oxidative stress induced by cigarette smoke exposure in guinea pigs affected by Zataria multiflora and its constituent, carvacrol. BMC Complement Altern Med.

[B16] Dinarello CA (2000). Proinflammatory cytokines. Chest.

[B17] Du K, Farhood A, Jaeschke H (2017). Mitochondria-targeted antioxidant Mito-Tempo protects against acetaminophen hepatotoxicity. Arch Toxicol.

[B18] Edenta C, Okoduwa SI, Okpe O (2017). Effects of aqueous extract of three cultivars of banana (Musa acuminata) fruit peel on kidney and liver function indices in wistar rats. Medicines (Basel).

[B19] Ezz-Eldin YM, Aboseif AA, Marwa M Khalaf MM (2020). Potential anti-inflammatory and immunomodulatory effects of carvacrol against ovalbumin-induced asthma in rats. Life Sci.

[B20] Farahpour MR, Sheikh S, Kafshdooz E, Sonboli A (2021). Accelerative effect of topical Zataria multiflora essential oil against infected wound model by modulating inflammation, angiogenesis, and collagen biosynthesis. Pharm Biol.

[B21] Ghorani V, Rajabi O, Mirsadraee M, Rezaeitalab F, Saadat S, Boskabady MH (2020). A randomized, doubled-blind clinical trial on the effect of Zataria multiflora on clinical symptoms, oxidative stress, and C-reactive protein in COPD patients. J Clin Pharmaco.

[B22] Hafez HM, Ibrahim MA, Ibrahim SA, Amin EF, Goma W, Abdelrahman, AM (2015). Potential protective effect of etanercept and aminoguanidine in methotrexate-induced hepatotoxicity and nephrotoxicity in rats. Eur J Pharmacol.

[B23] Hakimi Z, Salmani H, Marefati N, Arab Z, Gholamnezhad Z, Beheshti F, Shafei MN, Hosseini M (2020). Protective effects of carvacrol on brain tissue inflammation and oxidative stress as well as Learning and memory in lipopolysaccharide-challenged rats. Neurotox Res.

[B24] Hosseinzadeh H, Ramezani M, Salmani GA (2000a). Antinociceptive, anti-inflammatory and acute toxicity effects of Zataria multiflora Boiss extracts in mice and rats. J Ethnopharmacol.

[B25] Hosseinzadeh H, Ramezani M, Salmani G (2000b). Antinociceptive, anti-inflammatory and acute toxicity effects of Zataria multiflora Boiss extracts in mice and rats. J Ethnopharmacol.

[B26] Ischiropoulos H, Zh L, Beckman JS (1992). Peroxynitrite formation from macrophage-derived nitric oxide. Arch Biochem Biophys.

[B27] Jaeschke H, Farhood A, Cai SX, Tseng BY (2000). Protection against TNF-induced liver parenchymal cell apoptosis during endotoxemia by a novel caspase inhibitor in mice. Toxicol Appl Pharmacol.

[B28] Karimi S, Hosseinimehr SJ, Mohammadi HR, Khalatbary AR, Amiri FT (2018). Zatariamultiflora ameliorates cisplatin-induced testicular damage via suppression of oxidative stress and apoptosis in a mice model. Iran J Basic Med Sci.

[B29] Khazdair MR, Ghorani V, Alavinezhad A, Boskabady MH (2018). Pharmacological effects of Zataria multiflora Boiss L and its constituents focus on their anti-inflammatory, antioxidant, and immunomodulatory effects. Fundam Clin Pharmacol.

[B30] Kim EA, Kim SY, Ye BR, Kim J, Ko SC, Lee WW, Kim KN, Choi IW, Jung WK, Heo SJ (2018). Anti-inflammatory effect of Apo-9′-fucoxanthinone via inhibition of MAPKs and NF-kB signaling pathway in LPS-stimulated RAW 264 macrophages and zebrafish model. Int Immunopharmacol.

[B31] Mahmoodi M, Koohpeyma F, Saki F, Maleksabet A (2019). The protective effect of Zataria multiflora Boiss hydroalcoholic extract on TNF-α production, oxidative stress, and insulin level in streptozotocin-induced diabetic rats. Avicenna J Phytomed.

[B32] Mortazavi A, Mohammad Pour Kargar H, Beheshti F, Anaeigoudari A, Vaezi G, Hosseini M (2021). The effects of carvacrol on oxidative stress, inflammation, and liver function indicators in a systemic inflammation model induced by lipopolysaccharide in rats. Int J Vitam Nutr Res.

[B33] Nakhai LA, Mohammadirad A, Yasa N, Minaie B, Nikfar S, Ghazanfari G, Zamani MJ, Dehghan G, Jamshidi H, Boushehri VS (2007). Benefits of Zataria multiflora Boiss in experimental model of mouse inflammatory bowel disease. Evid Based Complement Alternat Med.

[B34] Nam Y, Ko SK, Sohn UD (2019). Hepatoprotective effect of ultrasonicated ginseng berry extract on a rat mild bile duct ligation model. J Ginseng Res.

[B35] Pawlotsky JM (2004). Pathophysiology of hepatitis C virus infection and related liver disease. Trends Microbiol.

[B36] Salmani H, Hakimi Z, Arab Z, Marefati N, Mahdinezhad MR, RezaeiGolestan A, Beheshti F, Soukhtanloo M, Mahnia A, Hosseini M (2022). Carvacrol attenuated neuroinflammation, oxidative stress and depression and anxiety like behaviors in lipopolysaccharide-challenged rats. Avicenna J Phytomed.

[B37] Sureshbabu A, Ryter SW, Choi ME (2015). Oxidative stress and autophagy: crucial modulators of kidney injury. Redox biol.

[B38] Sheibani V, Mandegary A, Vazifekhahan E, Kasbzade Z, Asadi A, Sharififar F (2019). Zataria multiflora Boiss extract improves spatial memory and learning capacity in scopolamine-induced amnesic rats. Avicenna J Phytomed.

[B39] Utaipan T, Suksamrarn A, Kaemchantuek P, Chokchaisiri R, Stremmel W, Chamulitrat W, Chunglok W (2018). Diterpenoid trigonoreidon b isolated from trigonostemon reidioides alleviates inflammation in models of lps-stimulated murine macrophages and inflammatory liver injury in mice. Biomed Pharmacother.

[B40] Wei L, Ren F, Zhang X, Wen T, Shi H, Zheng S, Zhang J, Chen Y, Han Y, Duan Z (2014). Oxidative stress promotes D-GalN/LPS-induced acute hepatotoxicity by increasing glycogen synthase kinase 3β activity. Inflamm Res.

[B41] Yakubu MT, Bilbis LS, Lawal M, Akanji MA (2003). Evaluation of selected parameters of rat liver and kidney function following repeated administration of yohimbine. Biokemistri.

[B42] Zhong W, Qian K, Xiong J, Ma K, Wang A, Zou Y (2016). Curcumin alleviates lipopolysaccharide induced sepsis and liver failure by suppression of oxidative stress-related inflammation via PI3K/AKT and NF-κB related signaling. Biomed Pharmacother.

